# Understanding subduction infancy to mature subduction in Southwest Japan via the self-consistent formation of a weak slab interface

**DOI:** 10.1038/s41598-023-48746-6

**Published:** 2023-12-05

**Authors:** Changyeol Lee, YoungHee Kim

**Affiliations:** 1 Department of Earth System Sciences, Yonsei University, 50 Yonsei–ro Seodaemun–gu, Seoul, 03722 Republic of Korea; 2 School of Earth and Environmental Sciences, Seoul National University, 1 Gwanak–ro, Gwanak–gu, Seoul, 08826 Republic of Korea

**Keywords:** Geodynamics, Seismology

## Abstract

The weak slab interface controls long-term subduction dynamics. A weak hydrous layer at the slab interface promotes mechanical decoupling between the forearc mantle and the subducting slab and converts a hot forearc mantle to a cold mantle. Often referred to as a cold nose, the cold forearc mantle, plays a key role in the transition from subduction infancy to mature subduction. This study was the first to numerically demonstrate the self-consistent formation of a weak hydrous layer with permeability anisotropy based on the Southwest Japan subduction zone case, where transition-related geological features were present. Our models showed that mechanical decoupling by spontaneous downdip growth of the weak hydrous layer created a cold nose by converting a hot forearc mantle to a cold mantle. The emergence of a cold nose explained the migration of the forearc-to-arc volcanic front, expressed as the formation of mid-Miocene forearc high-magnesium andesite and Quaternary arc adakite. Furthermore, the weak hydrous layer providing a pathway for free-water transport toward the mantle wedge tip elucidates slab/mantle-derived geochemical components in deep groundwater as well as large S-wave delay times and non-volcanic seismic tremors in the forearc.

## Introduction

Globally, one of the major observations in the forearc mantle of subduction zones is the anomalously cold corner of the mantle wedge (i.e., cold nose), as evidenced by low forearc heat flow^1,2^, a high P-to-S-wave velocity ratio^3^, and low seismic attenuation^4^. The presence of a cold nose is attributed to mechanical decoupling at the interface between the forearc mantle and subducting slab (i.e., slab interface), which extends down to a depth of ~ 70–80 km^1,5^. This decoupling prevents the sub-forearc mantle from participating in the corner flow of the mantle wedge. Thus, simulating the cold nose with numerical models has been an important subject, as the models can provide insight into time-varying subduction zone processes over geological time scales e.g^6–10^.

Recent advances in the detailed field- and laboratory-based observations^11,12^ have provided important insights into how mechanical decoupling by forming a weak hydrous layer develops at the slab interface over time, thereby improving our understanding of the transition from subduction infancy to mature subduction^11^. More specifically, a weak hydrous layer containing serpentinite and talc begins to develop via slab-derived fluids at the sub-forearc slab interface. The strain localization of such a layer simultaneously decouples the subducting slab from the adjacent sub-forearc mantle wedge, thus converting a hot forearc mantle to a cold nose (i.e., subduction infancy). The slab interface decoupled by this hydrous layer progressively deepens and finally reaches a downdip depth of ~ 80–100 km, with a sharp transition zone that extends for a few kilometers along the slab interface. The rheological switch from decoupling to fully viscous coupling at the downdip depth was formed within ~ 10–20 Myr after subduction initiation, thereby completing the formation of the cold nose (i.e., mature subduction).

Despite the importance of mechanical decoupling by the weak hydrous layer, a myriad of numerical models e.g^1,9,13–18^ has only provided a limited scope for the formation of such a layer because the ad hoc weakening implementation (e.g reduced subduction rate or imbedded weak layer at the slab interface) poses challenges for generating self-consistent layer formation. Only a few studies have considered the formation of a weak hydrous layer and its breakdown controlled by mineral phase diagrams, thus explaining the exhumation processes of high-pressure metamorphic rocks^19,20^, retreating/advancing trench migration^21,22^, seismic/petrologic observations^23,24^, and depth of mechanical decoupling^25^ in subduction zones.

**Figure 1:**
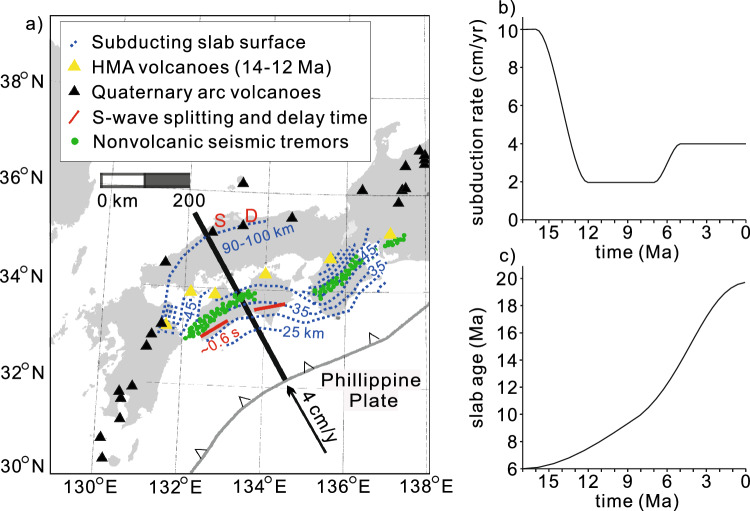
Geological and seismological observations in the Southwest (SW) Japan subduction zone. (**a**) A map showing major geophysical and geochemical events that occurred during the last 17 Ma, depicting the mid-Miocene high-magnesium andesite (HMA) and Quaternary arc (alkali-basalt and adakite) volcanoes, S-wave splitting direction and delay time, and non-volcanic seismic tremors. The red lines and texts below indicate fast polarization directions and delay times between the arrivals of the fast and slow S waves, respectively. A thick black line drawn from the trench indicates our model domain. Quaternary adakite erupted at the Sambe (S) and Daisen (D) volcanoes. All data plotted are cited in the text. (**b**) and (**c**) Subduction rate and slab age at the trench from 17 to 0 Ma ref^9,28^ used for the model experiments. Values are interpolated using a piecewise cubic spline. A panel **a** was adapted under CC BY-NC 4.0 from Fig. 1 ref^14^ using the GMT (Generic Mapping Tools; http://gmt.soest.hawaii.edu) software.

Similar to the Rio San Juan Complex in the Dominican Republic^19^, the Franciscan Complex in Northern California^26^, and other subduction zones^26,27^, the Southwest (SW) Japan subduction zone is a potential natural laboratory where the transition from subduction infancy to mature subduction via the formation of the weak hydrous layer can be inferred from a number of spatiotemporal observations after subduction initiation in ~ 17 Ma (Fig. 1). Observations included the migration of the forearc-to-arc volcanic front, expressed as the formation of mid-Miocene forearc high-magnesium andesite (HMA) (~ 15–12 Ma) and Quaternary arc adakite (< ~ 2 Ma)^28,29^. Geochemical features of volcanic rocks show that partial melting of the “wet” oceanic crust with and without garnet generates HMA at sub-forearc depth (< 70 km) and adakite at sub-arc depth (> 70 km), respectively^9,28,30^. The feature indicates that the forearc mantle was hot when HMA was formed; however, it cooled when adakite was formed beneath the arc. This phenomenon correlates well with the expansion and growth of the cold nose as the downdip depth of the weak hydrous layer increases. Furthermore, a weak hydrous layer below the cold nose can cause large S-wave delay times in the forearc mantle^31,32^.

Laboratory experiments show that the permeability anisotropy of foliated serpentinite in the weak hydrous layer results in a preferential flow of slab-derived fluids toward the mantle wedge tip^33,34^, thus explaining the slab/mantle-derived^3^He/^4^He values and associated ^20^Ne and Cl^−^ concentrations in deep groundwater in the forearc^35^, and deep non-volcanic seismic tremors in the forearc mantle^36^. To date, however, a quantitative understanding of such observations remains insufficient^9,14,15,28,29,33^. Although a recent numerical study^14^ considered the permeability anisotropy for generating the hydrous layer, it remains challenging to provide insight into how mechanical decoupling occurs because only ad hoc weakening conditions are imposed at the slab interface.

In this study, we employed two-dimensional (2-D) numerical models to consider the self-consistent formation of the weak hydrous layer with the permeability anisotropy of foliated serpentinite in the layer to explain spatiotemporal geological observations in SW Japan after subduction initiation at 17 Ma (see Methods for technical details of modeling). Under this numerical framework, our model successfully demonstrated the emergence of a weak hydrous layer at the slab interface that generated mechanical decoupling without a need for ad hoc constraint on the slab interface. This is the major advancement compared to the previous work of Lee and Kim^14^, providing important insights into how such layer can actively control subduction zone processes over geological time scales. Our model showed an emergence of this weak layer at ~ 15 Ma in SW Japan, consisting of hydrous minerals such as serpentine. Spontaneous downdip growth of the weak hydrous layer gradually decouples the subducting slab from the overlying mantle wedge, generating a cold nose, which explains the forearc to arc volcanic front migration. The free-water transport along the foliated serpentinite further clarifies present-day geophysical and geochemical observations in SW Japan. In summary, our model provides a unique opportunity to explore the 17-Myr-long evolution of the SW Japan subduction zone.

## Results

### Reference experiment

We first constrained subduction infancy in SW Japan after subduction initiation at 17 Ma (Fig. 1a). The model showed that the brucite in the lithospheric mantle, except for its top 6–7 km deep portion, broke down before reaching the depth of the mantle wedge due to the warm temperature of the ~ 6-Myr-old Philippine slab. Until ~ 15 Ma, the hot sub-backarc mantle intruded the corner of the mantle wedge as the overlying sub-forearc mantle was strongly coupled to the fast-subducting slab (~ 10 cm/yr) (i.e., an emergence of a hot corner of the mantle wedge). The subduction rate began to decrease at approximately 15 Ma and became 2 cm/yr at 12 Ma (Fig. 1b), in tandem with the decreased amount of bound water transported to the mantle wedge by the slab.

**Figure 2:**
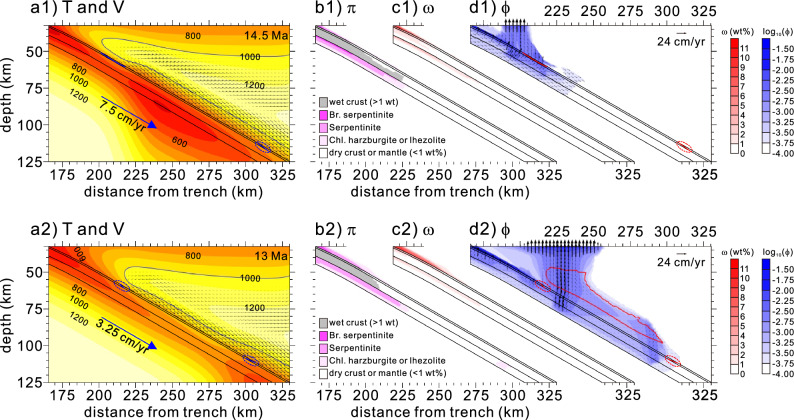
Model results obtained for 14.5 Ma (**a1–d1**) and 13 Ma (**a2–d2**). (**a1**) Distributions of temperature (T) and velocity (V) at 14.5 Ma. Black lines indicate boundaries of the basaltic oceanic crust, the gabbroic oceanic crust, and the hydrated portion of the lithospheric mantle. The magenta lines in the basaltic crust indicate wet basalt solidus fixed at 750 °C. The mantle velocity is normalized by the subduction rate. (**b1**) Distributions of major hydrous mineral phases (π) in the lithospheric mantle and “wet” basaltic and gabbroic oceanic crusts (bound water in hydrous minerals > 1 wt%) at 14.5 Ma. (**c1**) Distributions of the amount of bound water (ω) in the basaltic and gabbroic crusts and the hydrated portion of the lithospheric mantle and mantle wedge at 14.5 Ma. (**d1**) Distributions of free water (ϕ) and their velocities $\left( {{\mathop{V}\limits^{\rightharpoonup}} _{f} } \right)$ in the basaltic and gabbroic crusts and hydrated portions of the lithospheric mantle and mantle wedge at 14.5 Ma. The red contour line in the mantle wedge indicates the potential flux melting zone. (**a2**–**d2**) Same as above, except at 13 Ma.

At 14.5 Ma (subduction rate of ~ 7.5 cm/yr) (Fig. 2a1), as the hydrated portion of the slab reached the hot forearc mantle, basaltic and gabbroic oceanic crusts were continuously dehydrated at depths of ~ 35–50 km. The underlying lithospheric mantle (harzburgite) was dehydrated through breakdowns of the major hydrous minerals (π), such as brucite, serpentine, and chlorite, at depths of ~ 52, ~ 65–69, and ~ 70–75 km, respectively (Fig. 2b1,c1). Although the basaltic crust was fully dehydrated at depths of ~ 55–60 km, it was saturated by the free water expelled from the gabbroic crust and lithospheric mantle, and it was consequently partially molten (i.e., slab melting) at temperatures over the solidus of wet basalt (~ 750 °C) ref^37^. At a depth of ~ 110 km, melting of the basaltic crust did not occur because of the absence of free water. This feature was consistent with the formation of the mid-Miocene HMA volcanism in the forearc^28,29^. As the free water percolating through the wedge base transformed the dry mantle (lherzolite) into brucite-bearing serpentinite, serpentinite, or chlorite-bearing mantle, a weak hydrous layer began to form (Fig. 2c1,d1). Accordingly, the strain localization occurring in the layer decoupled the forearc mantle from the slab up to a depth of ~ 50 km (Supplementary Fig. 1a). Serpentinite foliation in the layer developed by strain localization provided a pathway for free-water transport toward the mantle wedge tip^33,34^ (Fig. 2d1).

Mechanical decoupling by the weak hydrous layer kept the slab interface cooler, which allowed for the deepening of mineral breakdown depth in the slab. At 13 Ma, the basaltic and gabbroic oceanic crusts were continuously dehydrated at depths of ~ 45–65 km (Fig. 2a2,b2). The lithospheric mantle was dehydrated through serpentine and chlorite breakdowns at depths of ~ 70–72 and ~ 72–77 km, respectively. The brucite in the lithospheric mantle was dehydrated at a depth of ~ 37.5 km before reaching the mantle wedge depth, as its dehydration was dominantly controlled by the decreased subduction rate instead of the temperature of the mantle wedge. The emergence of free water through mineral breakdowns at deeper depths hydrated the dry base of the mantle wedge, thus forming a weak hydrous layer at the slab interface.

Our model showed that the weak hydrous layer extended downward, and the downdip depth reached ~ 60 km (Fig. 2c2 and Supplementary Fig. 1b). Free water from the oceanic crust percolated through the weak hydrous layer toward the mantle wedge tip, and consequently, a small amount of free water entered the forearc mantle. The “free-water column” generated by the serpentine and chlorite breakdowns at depths of ~ 70–77 km in the lithospheric mantle saturated the sub-forearc basaltic crust, thus resulting in slab melting (> 750 °C), which can explain the HMA volcanism^28,29^ in the forearc (Fig. 2d2). The temperature of the basaltic oceanic crust also exceeded 750 °C at a depth of ~ 100 km, but negligible slab melting occurred because little free water liberated from the underlying lithospheric mantle may not saturate the oceanic crust. Thus, slab melting by free water saturation was limited to the forearc, consistent with the forearc HMA volcanism. Our model further constrained the potential flux melting zone, where the hydrated mantle temperature was sufficiently high for flux melting (bound water > 0.1 wt% and T > 1000 °C)ref^38^. The potential flux-melting zone developed above the slab appeared to be thin and narrow, and this spatially limited extent indicated less intense melting at the sub-forearc and arc mantle, consistent with the occurrence of sparse arc volcanism, except for the low alkali tholeiite related to the backarc opening^28,29^.

**Figure 3:**
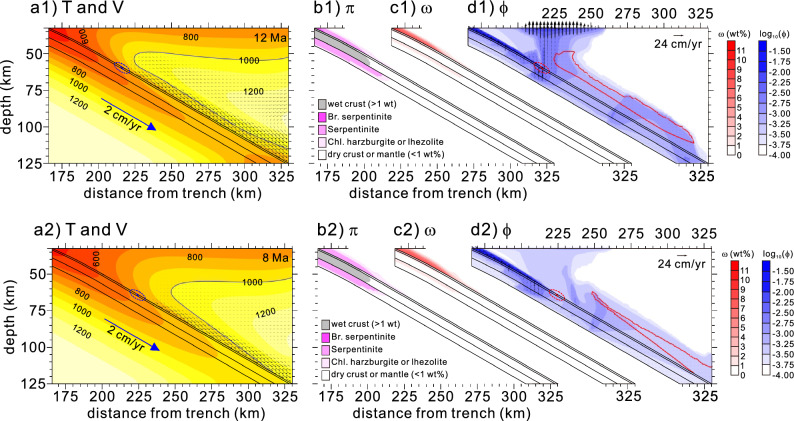
Model results obtained for 12 Ma (**a1–d1**) and 8 Ma (**a2–d2**). See Fig. 2 caption for detailed information.

At 12 Ma, as the subduction slowed down (2 cm/yr), slab dehydration took place at shallower depths; however, the downdip depth of the mechanical decoupling extended slightly deeper (Fig. 3a1–c1 and Supplementary Fig. 1c). In contrast to that observed at 13 Ma, the free-water column generated by serpentine and chlorite breakdowns at depths of ~ 65–70 km in the lithospheric mantle saturated the overlying sub-forearc basaltic crust. Nevertheless, only a small amount of slab melting may have occurred because the crustal temperature slightly exceeded the solidus temperature (> 750 °C). The decreased amount of free water and weakened corner flow due to slow subduction reduced the extent of the potential flux-melting zone (Fig. 3d1).

Since ~ 12 Ma, a lower portion of the weak hydrous layer had been dragged by the subducting slab e.g^14^, thus resulting in the extension of the downdip depth of the mechanical decoupling (e.g., 8 Ma; Supplementary Fig. 1d). The free-water column from the lithospheric mantle still saturated the sub-forearc basaltic crust. However, slab melting did not occur because the temperature of the basaltic crust was still lower than the solidus temperature (< 750 °C) (Fig. 3a2–d2). Most of the free water originating from the slab was transported toward the mantle wedge tip through the weak hydrous layer; much less free water reached the mantle wedge, thus resulting in reduced potential flux melting. Features such as the slow extension of the downdip depth of the mechanical decoupling, absence of slab melting, and reduced potential flux melting that persisted until ~ 4 Ma are consistent with the scattered monogenetic alkali basalt forearc and arc magmatism from ~ 12 to 4 Ma ref^28,29^.

**Figure 4:**
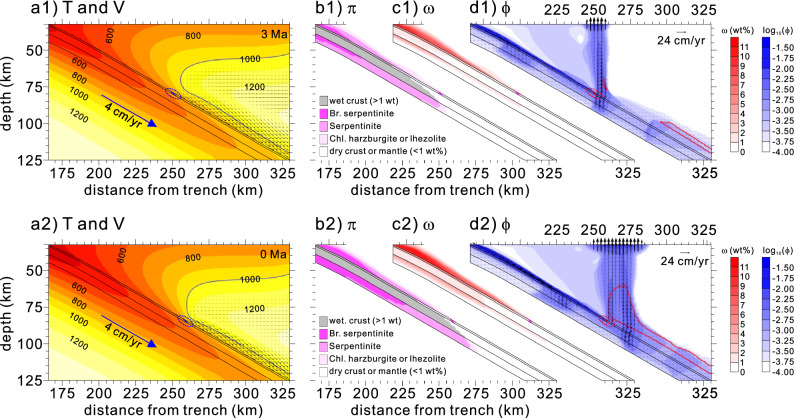
Model results obtained for 3 Ma (**a1–d1**) and 0 Ma (present-day) (**a2**–**d2**). See Fig. 2 caption for detailed information.

As the subduction rate increased from 2 cm/yr at 7 Ma to 4 cm/yr at 5–0 Ma, our model showed increases in the amount of bound water and breakdown depth (Fig. 4a1–c1), whereas the absence of slab melting and reduction of potential flux melting continued until ~ 4 Ma. Because the weak hydrous layer was dragged further at a higher rate, the downdip depth of the mechanical decoupling extended to a depth of ~ 80 km at 3 Ma (Fig. 4d1 and Supplementary Fig. 1e). Although much of the free water liberated from the slab percolated through the weak hydrous layer toward the mantle wedge tip, a little free water liberated from the lithospheric mantle resumed partial melting of the overlying basaltic crust (at > 750 °C and a depth of  > 70 km), forming adakite instead of HMA (Fig. 4b1–d1). At similar depths, mineral breakdowns in the dragged weak layer (i.e., chlorite-bearing mantle) provided free water to the mantle wedge; however, the degree of flux melting in the mantle was expected to be weak, expressed as a small potential flux melting zone. Over time, the downdip depth of the mechanical decoupling extended deeper and reached ~ 85 km by 0 Ma (Fig. 4a2 and Supplementary Fig. 1f.), completing the formation of the cold nose in the matured SW Japan subduction zone. The chlorite in the weak hydrous layer and slab broke down at the downdip depth and at a depth of ~ 100 km, respectively (Fig. 4b2,c2). Free water liberated from chlorite breakdown resulted in both sub-arc slab and flux melting (> 750 °C and 1000 °C, respectively), identified by adakite and arc-type alkali-basalt volcanism, respectively^28,29^. Although a greater amount of free water existed at the base of the sub-backarc mantle wedge than at 3 Ma (Fig. 4d2), relatively small amounts of bound and free water in the wedge base may yield weak flux melting in the backarc, which could explain the dominant rift-type volcanism caused by decompression melting instead of arc-type volcanism caused by flux melting^28,29^.

### Parametric study: role of permeability anisotropy in the formation of a weak hydrous layer

Serpentinite is a major component of the weak hydrous layer in our model. A wide range of uncertainties in the serpentinite permeability was observed in laboratory experiments^33^ as it is typically extrapolated by considering the deep mantle environment. However, a recent study^39^ provided serpentinite permeability directly measured under mantle pressure conditions (up to 3 GPa), approximately one order of magnitude lower than that used in our model. To account for such large variations in serpentinite permeability, we evaluated the effect of the permeability anisotropy of serpentinite by using low and high permeability values, corresponding to 0.1 and 12.5 times that used for our reference experiment, respectively. Results showed that with the high permeability value, the permeability parallel to the foliation is equivalent to the isotropic peridotite permeability (*K*_*m*_); with the low permeability, the permeability normal to the foliation becomes 0.02 of *K*_*m*_. All the other model parameters and rheology were the same as those used in the reference experiment.

**Figure 5:**
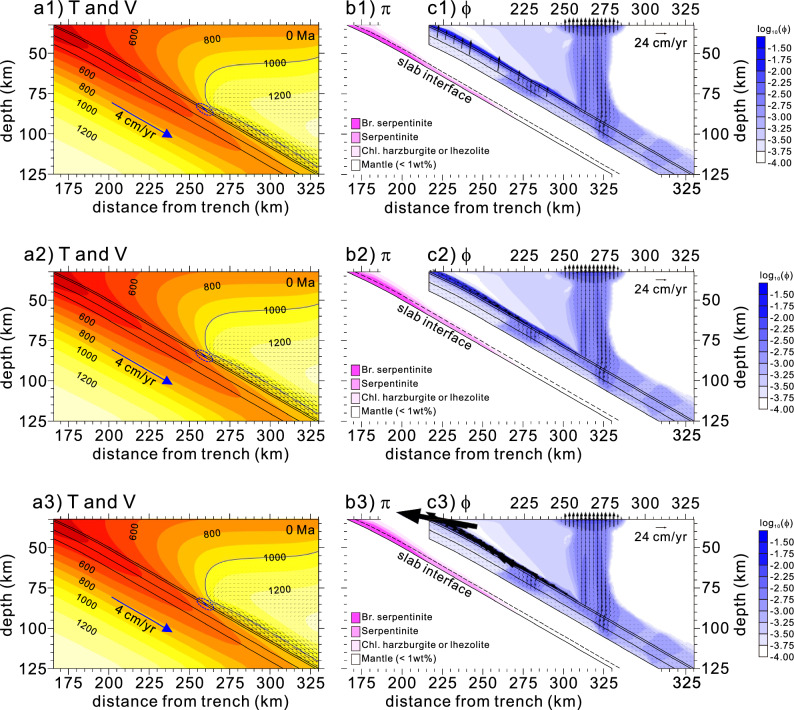
Distributions of the weak hydrous layer at the slab interface and free water and velocity obtained from the experiments for 0 Ma (present-day) with varying serpentinite permeability. (**a1–a3**) Distributions of temperature (T) and velocity (V) from the model experiments using low (0.1 times that of the reference experiment), reference, and high permeability values of serpentinite (12.5 times that of the reference experiment). (**b1–b3**) Distributions of major hydrous mineral phases (π) in the lithospheric mantle and “wet” basaltic and gabbroic oceanic crusts (bound water in hydrous minerals > 1 wt%). A dashed line, 2.5 km above the slab interface, is a visual aid to measure the thickness of the weak hydrous layer. (**c1–c3**) Distributions of free water (ϕ) and their velocities $\left( {{\mathop{V}\limits^{\rightharpoonup} }_{f} } \right)$ in the basaltic and gabbroic crusts and hydrated portions of the lithospheric mantle and mantle wedge. See Fig. 2 caption for detailed information.

Our models demonstrated that a cold nose forms regardless of variations in serpentinite permeability. However, increased serpentinite permeability elevates both the thickness of the weak hydrous layer and free-water velocity within the layer (Fig. 5). The experiment using low serpentinite permeability showed a thinner weak hydrous layer at the slab interface (1.96-km-thick serpentinite layer at a depth of 60 km) than those from the reference value and high permeability (3.01- and 3.25-km-thick serpentinite layers at a depth of 60 km, respectively) (Fig. 5b1 vs. b2 and b3). The low permeability allowed for the slow growth of the weak hydrous layer, normal to foliation (Fig. 5b1 vs. b2 and b3). The low permeability of the weak hydrous layer further kept free water accumulated in the basaltic oceanic crust beneath it, extending to a depth of ~ 75 km (Fig. 5c1). In contrast, the weak hydrous layer thickened as the permeability increased, and free water quickly escaped from the slab through the weak hydrous layer (Fig. 5c2 and c3). Thus, the amount of the free water escaped across the Moho increases with serpentinite permeability: ~ 0.38, ~ 16.9, and ~ 191.7 ton/yr per 1 m along the trench (Fig. 5c1–c3, respectively). Although the free-water velocity and layer thickness varied depending on the permeability values, our models exhibited the formation of a cold nose that shows the transition from subduction infancy to mature subduction.

**Figure 6:**
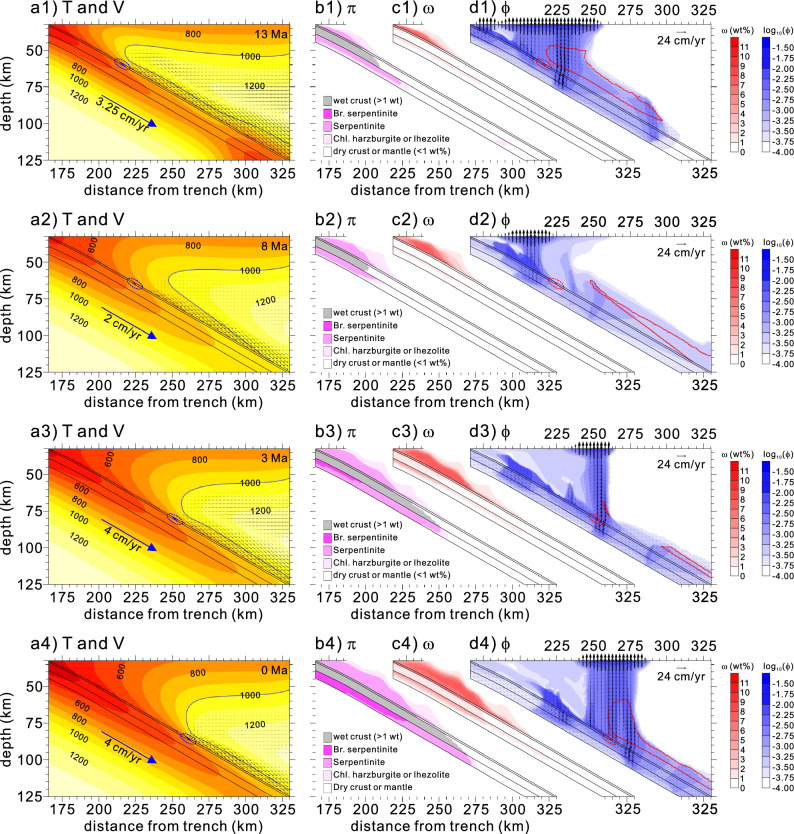
Model results obtained for 13 (**a1**–**d1**), 8 (**a2**–**d2**), 3 (**a3**–**d3**), and 0 Ma (present-day) from the model experiment using the isotropic serpentinite permeability. See Fig. 2 caption for detailed information.

To assess the effectiveness of permeability anisotropy in generating a weak hydrous layer, we conducted a model experiment using the isotropic serpentinite permeability. The assigned permeability value (0.08 km) normal to foliation was the same as that parallel to foliation used in the reference experiment. No other parameters or rheology were changed. The model demonstrated a similar style for the formation of a weak hydrous layer after subduction initiation to the results of the reference experiment (Fig. 6). The model further demonstrated that the hot corner of the mantle wedge was converted to a cold nose over time, similar to the features shown in the reference experiment. The major difference between the model and reference experiments was that the free water originating from the subducting slab no longer traveled through the weak hydrous layer toward the mantle wedge tip. Lastly, we used peridotite permeability to examine its effect on the formation of the weak hydrous layer. We observed that the result was nearly identical to that observed in the model experiment using the isotropic serpentinite permeability (not shown).

## Discussion

**Figure 7:**
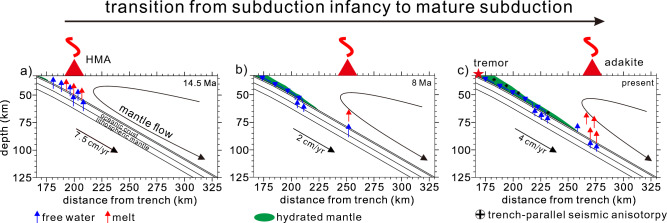
Schematic descriptions of the major geophysical and geochemical observations correlated with the formation of a weak hydrous layer at the slab interface in the SW Japan subduction zone. Hydrated portions of the subducting slab can be obtained from the Supplementary Fig. 3a. (**a**) During the early stage of the subduction (e.g., 14.5 Ma), slab dehydration mainly occurs at the sub-forearc slab and results in slab melting, explaining the high-magnesium andesite (HMA) in the forearc. Blue and red arrows correspond to the flow directions of the free water and melt, respectively. The green zone indicates the hydrated mantle, which forms the weak hydrous layer at the slab interface. (**b**) Ongoing formation of a weak hydrous layer at the slab interface decouples the mantle from the subducting slab toward the arc. The foliated serpentinite in the hydrous layer detours the free water toward the mantle wedge tip. The less slab-derived free water due to the slow subduction results in less flux melting, forming the scattered monogenetic forearc and arc magmatism. (**c**) The present distribution of the weak hydrous layer at the slab interface detours the free water to the mantle wedge tip, explaining the non-volcanic seismic tremor, trench-parallel seismic anisotropy (S-wave splitting direction), and groundwater geochemistry in the forearc. The slab melting by the slab-derived free water from the underlying lithosphere results in the adakite in the arc.

Our model captured the essential features of the transition of subduction from infancy to maturity in SW Japan since 17 Ma (Fig. 7). The spontaneous emergence of a weak hydrous layer and its downdip growth facilitated mechanical decoupling between the subducting slab and overriding mantle wedge, thus converting a hot forearc mantle into a cold nose. We note that these features were presumed in the numerical model of our previous study^14^; hence, they cannot systematically evaluate the spatiotemporal evolution of the features. Our model provided a quantitative assessment of forearc-to-arc volcanic front migration, contemporary with the development of the mid-Miocene HMA and Quaternary adakite, which has been only qualitatively suggested in previous studies^28,29^.

The low permeability of the lower continental crust (two orders of magnitude less permeable than that of serpentinite) above the sub-forearc serpentinite layer allows for fluid accumulation and overpressure at or near the mantle wedge tip and at the continental Moho^34^, possibly leading to the spatiotemporal distribution of slow earthquakes (including tremors and slow slips) in SW Japan^40^. Sparse arc volcanism^28,29^ and slab/mantle-derived geochemical components in deep groundwater^35^ can be evidence to confirm free-water transport toward the mantle wedge tip along the weak layer in SW Japan. The weak hydrous layer can further explain the high degree of anisotropy observed as the large delay times between two split S waves^31,32^ (Fig. 7c). All features can be explained when the permeability anisotropy of the foliated serpentinite exists in the weak layer.

Our experiment using the low permeability of the weak hydrous layer showed a small amount of free water escaped across the Moho; however, an excess amount of free water was observed along the basaltic oceanic crust of the subducting slab at a depth of ~ 75 km (Figs. 5a1–c1), which suggested overpressurized fluid in the oceanic crust^39^. However, the overpressurized fluid in the oceanic crust may not extend down to such depths because the tremor locations in SW Japan are mostly concentrated at or near the mantle wedge tip and continental Moho^36^; this indicated that the permeability of the weak hydrous layer might not be lower than that used in the reference experiment.

Because our experiments did not consider the effect of dynamic pressure on free-water transport, unlike several previous studies^7,41^, we separately evaluated the effect of the dynamic pressure of the solid flow on free-water transport (see the Supplementary Information for details). The model calculations showed a negligible impact of the dynamic pressure on the formation of the weak hydrous layer toward the mantle wedge tip, thus demonstrating that permeability anisotropy was indeed the key factor.

Wilson et al^42^., considering the compaction pressure in their numerical models, developed two pathways for free water in the shallow portions of the subducting slab and mantle wedge, which deflected the moving direction of free water from the sub-backarc to the sub-arc depth. However, the effect of compaction pressure on our model calculations would be minor because most slab dehydration was completed at sub-arc depth. Hence, there is only a small amount of free water in the sub-backarc mantle, and the slab was influenced by compaction pressure (Figs. 2, 3, 4, 5 and 6). In addition, free water percolating through the foliated serpentinite in the weak hydrous layer was less influenced by the compaction pressure because the compaction length of the low-viscosity layer was even shorter than that of the overlying mantle. The viscous resistance to volume changes was relatively small^43^. In contrast, Wang et al^44^. considered the compaction pressure and dynamic response of the overlying crust. They showed that bifurcated slab-derived free water percolates toward the corner of the mantle wedge. This may provide more free water to the mantle tip wedge through foliated serpentinite in the weak hydrous layer. This indicated that considering the compaction pressure and dynamic response of the overlying crust would not significantly change our findings.

Our model captured a feature showing that the downdip depth of mechanical decoupling, controlled by pressure and temperature conditions, increased slowly over time as the subducting slab dragged a lower portion of the weak hydrous layer. This phenomenon explained the forearc-to-arc volcanic front migration, contemporaneous with the development of the mid-Miocene HMA and Quaternary adakite^28,29^, which has not been explained by the previous model of Lee and Kim^14^ with ad hoc mechanical weakening at the slab interface. Previous studies^6,7^ demonstrated that a hydrous layer formed at the sub-forearc slab interface by slab-derived free water was dragged by the subducting slab and broken down by increased pressure and temperature, similar to what was observed in our model. However, using the same constant value, these studies oversimplified the viscosities of the mantle and weak hydrous layer. The hydrous layer was formed due to the low temperature of the thermal boundary layer at the slab surface instead of mechanical decoupling by strain localization, as shown in our model (Supplementary Fig. 1). The downdip depth of mechanical decoupling may be limited to a particular depth of ~ 80 km because, beyond this depth, the weak hydrous layer may detach from the subducting slab due to its positive buoyancy and enter the overlying mantle^45,46^. If the self-regulation of mechanical decoupling by the weak hydrous layer was considered, the transition from subduction infancy to mature subduction in SW Japan could be more precisely constrained.

The subduction rate of the Philippine Plate has largely varied since 17 Ma (Fig. 1b); however, the mechanism of its variation remains poorly understood. Hence, the subduction rate was kinematically considered in our model experiments. Behr and Becker^13^ suggested that a mechanically weak sedimentary layer at the slab surface reduces the viscous stress at the slab interface, thus increasing the subduction rate. The formation of a weak hydrous layer at the slab surface, which occurs a few million years after subduction initiation in our model, can reduce the viscous stress at the slab interface. Therefore, the observed abrupt decrease in subduction rate may be caused by strong mechanical coupling with the warm (buoyant) slab at the interface because of the absence of a weak hydrous layer, and its increase was then significantly linked to the formation of a weak hydrous layer and increased negative buoyancy of the slab (i.e., slab pull) over time (Fig. 1c). A model using a dynamically driven subducting slab could constrain the role of the formation of a weak hydrous layer in the evolution of the subduction rate in SW Japan.

Our study on the self-consistent formation of the weak hydrous layer at the slab surface can potentially explain the Quaternary adakite^47^ and slow earthquakes^48^ in Cascadia, despite the fact that its subduction stage is near the end (i.e., ridge subduction) compared to the maturity of the SW Japan subduction zone. Although this study sheds light on the inference that the formation of a weak hydrous layer is an effective mechanical decoupling mechanism, mechanical decoupling in old subduction zones remains unresolved because there is no clear observation of such a layer at the sub-forearc slab interface^49^ (e.g., Northeast Japan). Hence, the weak hydrous layer can be too thin to be seismologically detected^50^, and/or undiscovered processes can occur at the slab surface^49^.

## Summary

Although a sub-forearc weak hydrous layer at a slab interface plays a critical role in subduction zone processes over geological time scales, the transition from subduction infancy to mature subduction has not been evaluated in numerical models due to methodological issues related to the modeling. This study aimed to numerically demonstrate the self-consistent formation of a weak hydrous layer with permeability anisotropy, which enabled mechanical decoupling by spontaneous layer formation. Moreover, this study was the first to examine the transition based on the case of the SW Japan subduction zone. Our numerical observations provide useful insight into notable geological observations, such as forearc-to-arc volcanic front migration after subduction initiation and present-day geophysical and geochemical observations in the forearc.

## Methods

### Governing equations

We constructed a 2-D finite element model that included a kinematically driven subducting slab, a dynamically driven mantle wedge, and a stationary overlying continental crust (Fig. 1 and Supplementary Fig. 3). Therefore, only the energy equation was solved for the subducting slab and continental crust while the incompressible continuity, Stokes, and energy equations were solved for the mantle wedge:

Continuity equation,

1 $$0 = \nabla \cdot {\mathop{V}\limits^{\rightharpoonup}} _{s}$$

Momentum equation,

2 $$0=-\nabla P+\nabla \cdot (2\eta \dot{\varepsilon })$$

Energy equation,

3 $$\rho_{s} C_{p} \left( {\frac{\partial T}{{\partial t}} + {\mathop{V}\limits^{\rightharpoonup}} _{s} \cdot \nabla {\text{T}}} \right) = \nabla \cdot (k\nabla {\text{T )}} + {\text{H}}$$

where ${\mathop{V}\limits^{\rightharpoonup}}_{s}$ is the solid velocity; $P$ is the dynamic pressure; $\eta$ is the shear viscosity; $\dot{\varepsilon }$ is the strain rate tensor; ${\rho }_{s}$ is the solid (rock) density; ${C}_{p}$ is the specific heat;* T* is the temperature; *t* is the time; $k$ is the thermal conductivity; and *H* is the radiogenic heat production. The net mantle adiabat ($\overline{T }$) was posteriorly added to the calculated temperatures e.g^1, 5, 14^. Because the behavior of the mantle wedge was dominated by corner flow, thermal and compositional buoyancies were neglected.

Compared with our previous model^14^, this model incorporated the rheology of weak hydrous minerals (e.g., serpentine) without imposing ad hoc weakening at the slab interface. The serpentine viscosity (e.g., antigorite) was over three orders of magnitude lower (~ 10^−18^–10^19^ Pa s) than that of olivine under sub-forearc mantle pressure and temperature conditions^51^. Moreover, only 10–15% serpentinization of the peridotite showed rheology similar to that of serpentinite^52^. Viscosity of nominally anhydrous olivine significantly decreases with increased amount of bound water though such relation has not been quantitatively evaluated yet. We thus approximated the viscosity of bound-water bearing olivine by multiplying the viscosity reduction factor to the calculated viscosities of diffusion and dislocation creep of dry olivine^53^; the viscosity reduction factors were expressed as functions of the amount of bound water e.g^55^. In the mantle wedge, dislocation creep dominates the mantle rheology^53^. Thus, viscosity reduction in the dislocation-creep equation was set less sensitive to the amount of bound water than that of diffusion creep to comply with the estimated viscosity of the upper mantle^53,59^. We further assumed that the viscosities of olivine and serpentine represented the viscosities of mantle and serpentinite, respectively. To approximate the viscosity of the mantle wedge by considering all the rheologies described above, we used the composite viscosities of olivine and serpentine as follows:

Composite viscosity,

4 $$\eta ={\left(\frac{1}{{\eta }_{ol, dif}}+\frac{1}{{\eta }_{ol,dis}}+\frac{1}{{\eta }_{serp,dis}}\right)}^{-1}$$

Diffusion creep of olivine,

5 $${{\eta }_{ol,dif}=\left(\frac{{\omega }_{r}}{\omega }\right)\frac{\mu }{2{A}_{ol,dif}}\left(\frac{b}{d}\right)}^{-m}exp\left(\frac{{E}_{ol,dif}+P{V}_{ol,dif}}{R(T+\overline{T })}\right)$$

Dislocation creep of olivine,

6 $${\eta }_{ol,dis}=\left(\frac{3{\omega }_{r}}{\omega +{2\omega }_{r}}\right)\frac{\mu }{2}{A}_{ol,dis}^{-\frac{1}{{n}_{ol}}}exp\left(\frac{{E}_{ol,dis}+P{V}_{ol,dis}}{{n}_{ol}R(T+\overline{T })}\right){\dot{\varepsilon }}^{\frac{1-{n}_{ol}}{{n}_{ol}}}$$

Dislocation creep of serpentine

7 $${\eta }_{serp,dis}=f(\omega )\frac{1}{2}{A}_{serp,dis}^{-\frac{1}{{n}_{serp}}}exp\left(\frac{{E}_{serp,dis}+P{V}_{serp,dis}}{{n}_{serp}R(T+\overline{T })}\right){\dot{\varepsilon }}^{\frac{1-{n}_{serp}}{{n}_{serp}}}$$

where the subscripts *ol*, *serp*, *dif*, and *dis* refer to olivine, serpentine, diffusion creep, and dislocation creep, respectively; *ω* is the mass fraction of bound water; *ω*_*r*_ is the background mass fraction of bound water for dry olivine; *μ* is the shear modulus;* A* is the pre-exponential factor; *b* is the Burgers vector; *d* is the grain size; *m* is the grain size exponent; $n$ is the stress exponent; *E* and *V* are the activation energy and volume, respectively; *R* is the gas constant; and *f* ($\omega$) is a function of bound water for activating the serpentine viscosity and becomes unity when 20% or more of the peridotite is serpentinized ($\omega$: 2.153 wt%). If the amount of bound water is small ($\omega$ < 0.15 wt%), the olivine viscosity reduced by the increased bound water dominates mantle viscosity^54,55^. If the amount of bound water is large ($\omega$ > 2.153 wt%)*,* the serpentine viscosity dominates the mantle viscosity. Although the viscosity of the chlorite generated by serpentine breakdown is not adequately constrained, coexisting talc significantly reduces the viscosity of chlorite-bearing peridotite^56^. Thus, the viscosity of the chlorite-bearing peridotite was calculated using the composite viscosity equation. This rheology setup allows for a substantially low viscosity in the sub-forearc mantle, where hydrous minerals are present.

The bound water in the minerals is simultaneously transported by the subducting slab and the convecting mantle wedge. In contrast, the free water in the pores is transported by porous flow in the deforming solid. Using the zero-compaction length and small-porosity approximations, the bound- and free-water transport can be calculated as follows^14,15,18,42,57,58^:

Bound-water transport,

8 $$\frac{\partial \omega }{{\partial t}} + \nabla \cdot \left( {{\mathop{V}\limits^{\rightharpoonup}} _{s} \omega } \right) = \Gamma_{f \to s} /\rho_{s} + \nabla \cdot (\kappa_{\omega } \nabla \omega {)}  $$

Free-water transport,

9 $$\frac{\partial \phi }{{\partial t}} + \nabla \cdot \left( {{\mathop{V}\limits^{\rightharpoonup}} _{f} \phi } \right) = \Gamma_{s \to f} /\rho_{f} + \nabla \cdot (\kappa_{\phi } \nabla \phi {)}$$

where $\phi$ is the volume fraction of free water (i.e., porosity); ${\mathop{V}\limits^{\rightharpoonup}}_{f}$ is the free-water velocity; $\Gamma_{f \to s}$ and $\Gamma_{s \to f}$ are the rates of mass transfer by hydration and dehydration, respectively; ${\rho }_{s}$ and ${\rho }_{f}$ are the solid and free-water densities, respectively; and ${\kappa }_{\omega }$ and ${\kappa }_{\phi }$ are the artificial diffusivities of bound and free water, respectively, for the diffusion terms to improve the model stability without significantly influencing the water transport^18^. The free-water velocity, assumed to be controlled by the solid flow and fluid buoyancy, can be calculated as follows^14,15^:

 Free-water velocity,

10 $${\mathop{V}\limits^{\rightharpoonup}}_{f} = {\mathop{V}\limits^{\rightharpoonup}}_{s} - \frac{{\mathbf{K}}}{{\eta_{f} \phi }}\left( {\nabla P - \Delta \rho } \right)\mathop{g}\limits^{\rightharpoonup}$$

where $\mathbf{K}$ is the permeability tensor (i.e., ${\mathbf{K}}_{{\varvec{i}}{\varvec{j}}}$, *i* and *j* for the x- and y-axes, respectively); ${\eta }_{f}$ is the free water viscosity; $\Delta \rho$ is the density difference between the solid and free water (${\rho }_{s}-{\rho }_{f}$); and $\mathop{g}\limits^{\rightharpoonup}$ is the gravitational acceleration. Because the effective viscosity of the mantle wedge is relatively small^59^, the impact of dynamic pressure on free-water transport may be minor. Thus, the dynamic pressure term was neglected here and was evaluated separately (see Supplementary Information for details).

The isotropic peridotite permeability (*K*_*m*_) is approximated as that of a texturally equilibrated rock^60^:

Isotropic permeability,

11 $${K}_{m}=\frac{{d}^{2}{\phi }^{3}}{{C}_{d}}$$

where ${C}_{d}$ is the geometrical factor. Using a background volume fraction of free water (${\phi }_{0}$ = 10^−4^) and a volume fraction of 10^−3^, we obtained a reference peridotite permeability (*K*_*0*_) of 1.481 × 10^−20^ and a permeability of 1.481 × 10^−17^ m^2^, respectively. Previous studies^61,62^ showed that the decreased rock permeability with increased depth (pressure) asymptotically converges to a certain value at a depth of ~ 30 km. Thus, we neglected the effect of pressure on mantle permeability.

The permeability along the serpentinite foliation was approximately two orders of magnitude higher than that normal to foliation, though it was still significantly (~ 1–2 orders) lower than the isotropic peridotite permeability^33^. When serpentinite formed, strain localization due to its low viscosity led to foliation parallel to the slab interface, creating a pathway for free-water transport toward the mantle wedge tip (i.e., serpentinite channel)^11,33,50^. Thus, the permeability tensor of serpentinite, which can be expressed as a function of the foliation orientation ($\theta$) from the reference frame (x-axis), is defined as follows^63,64^:

Permeability tensor of serpentinite,

12 $$\mathbf{K}=\left(\begin{array}{cc}{K}_{n}{sin}^{2}\theta +{K}_{p}{cos}^{2}\theta & ({K}_{n}-{K}_{p})sin\theta cos\theta \\ ({K}_{n}-{K}_{p})sin\theta cos\theta & {K}_{n}{cos}^{2}\theta +{K}_{p}{sin}^{2}\theta \end{array}\right)$$

where *K*_*n*_ and *K*_*p*_ correspond to the permeabilities normal and parallel to the foliation, defined as 0.0016 km and 0.08 km, respectively. The 10–15% serpentinization of the peridotite develops a network of weak serpentine^52^; thus, the permeability anisotropy of the serpentinite channel was assumed to be activated when 20% of the peridotite was serpentinized.

The (de)hydration of the oceanic crust and mantle was considered using a simplified kinetic relationship formulated as follows^14,57,58^:

Mass transfer from free to bound water,

13 $$\Gamma_{f \to s} = \frac{{\rho_{s} \left| {{\mathop{V}\limits^{\rightharpoonup}} _{s} } \right|\left( {\omega_{{max\left( {P,T + \overline{T}} \right)}} - \omega_{{\left( {P,T + \overline{T}} \right)}} } \right)}}{l}$$

Mass transfer from bound to free water,

14 $$\Gamma_{s \to f} = - \Gamma_{f \to s}$$

where $\left| {{\mathop{V}\limits^{\rightharpoonup}} _{s} } \right|$ is the magnitude of the solid velocity; ${\omega }_{max\left(P,T+\overline{T }\right)}$ is the maximum mass fraction (solubility) of bound water at a given pressure and temperature; ${\omega }_{\left(P,T+\overline{T }\right)}$ is the mass fraction of bound water; and *l* is the (de)hydration length, sufficiently short for the rapid (de)hydration of the oceanic crust and mantle without creating a metastable phase. The parameter values used in this study are listed in Supplementary Table 1.

### Model setup

From ~ 30 to 17 Ma, SW Japan was the margin of the Eurasian plate and bounded by a transform fault between the margin and the Shikoku Basin^28^. Among the diverse mechanisms of subduction initiation^65–67^, subduction of the Philippine plate beneath SW Japan was induced by a backarc opening at ~ 17 Ma ref^9,28^. To consider such subduction history, we constructed a 2-D model (329.9 km long and 125 km deep) along the trench-perpendicular profile in the SW Japan subduction zone (Fig. 1 and Supplementary Fig. 3). The model included 100-km- and 32.5-km-thick subducting slabs and overlying continental crust, respectively, with a sandwiched mantle wedge along the profile. To solve the Stokes equation, only applicable to the mantle wedge, the subduction rate was set at the slab interface (Supplementary Fig. 3). To minimize the pressure singularity at the mantle wedge tip, a 1-km-long linear velocity ramp was imposed at the slab interface from the tip^10^; no arbitrary decoupling zone was prescribed at the slab interface except for the tip. No-slip and open boundaries were set for the continental Moho and outer boundary of the mantle wedge, respectively. A zero velocity was set as the initial velocity of the mantle wedge. To solve the energy equation, the top surface temperature of the model domain was fixed at 0 °C, and the bottom of the subducting slab was insulated. The horizontal boundary of the subducting slab at a depth of 125 km was prescribed as an open boundary. The half-space cooling model for a 40-Myr plate was calculated using a mantle potential temperature of 1350 °C, and the calculated temperature profile was prescribed to the backarc-side vertical boundary of the mantle wedge at a depth of 100 km. For the initial temperature, the temperature profile calculated from the half-space cooling model for the 40-Myr plate was assigned to the model domain, consistent with the normal mantle temperature without subduction from ~ 30 to 17 Ma ref^28^. A net mantle adiabat of 0.35 °C/km was added to the calculated temperatures as a posterior. Radiogenic heat production from tholeiitic basalt, granite (half value), and peridotite was assigned to the oceanic crust, continental crust, and mantle, respectively^68^. The time-evolving subduction rate and slab age constrained from plate reconstruction after subduction initiation in 17 Ma ref^9,28^ (Fig. 1b,c) were assigned to the subducting slab and trench-side vertical boundary, respectively, via the half-space cooling model.

Although the amount and depth extent of the bound water in the subducting slab were not well constrained, outer-rise faults developed in the subducting slab prior to subduction allowed for hydration of the oceanic crust and upper portion of the underlying lithospheric mantle^69,70^. The hydrated portion of the slab consisted of a 1-km-thick basaltic oceanic crust layer, a 5-km-thick gabbroic oceanic crust layer, and a 5-km-thick residual harzburgite layer (Supplementary Figs. 3a, 3b, and Supplementary Table 2). The sedimentary layer was neglected because the layer thickness subducted into the mantle was only ~ 0.3 km ref^71^, which yielded an insignificant contribution to the bound-water budget, except for a large contribution to the geochemical components^72^. The relevant phase diagrams showing the water solubilities (i.e., maximum bound water) of the three layers were evaluated using Perple_X software (ver. 6.8.6) with the embedded activity models (Supplementary Table 2) and a Na_2_O–CaO–K_2_O–MgO–FeO–MnO–Al_2_O_3_–TiO_2_–SiO_2_–H_2_O–CO_2_ system^73^. The maximum water solubilities of basalt, gabbro, and harzburgite were 5.03802, 5.01913, and 10.7652 wt%, respectively^72,74^ (Supplementary Figs. 4a–c). To consider the depth-dependent and localized bound-water distribution along the faults^69,70^, each layer was divided into 1-km-thick sublayers. Different degrees of hydration were prescribed: 100% hydration for the basaltic oceanic crust at a depth of 0–1 km, 50%, and 25% hydration for the gabbroic oceanic crust at depths of 1–2 km and 2–6 km, respectively, and lastly 40, 30, 20, 10, and 5% hydration for the residual harzburgite at depths of 6–7, 7–8, 8–9, 9–10, and 10–11 km, respectively. These values indicated that phase diagrams controlled the dehydration of the oceanic crust and lithospheric mantle; however, the amount of expelled free water was reduced according to the degree of hydration. The expelled free water hydrated the overlying mantle wedge (lherzolite), whose water solubility was calculated using the depleted MORB mantle^72^ (Supplementary Fig. 4d). The relevant amounts of bound water for the hydrated upper portion of the subducting slab were assigned to the trench-side vertical boundary, and the background mass fraction of bound water (0.01 wt%) was set at the outer boundary of the mantle wedge. The initial mass fraction of the bound water was 10^−4^ (0.01 wt%). No bound-water transport was allowed across the inner and outer boundaries to prevent artificial diffusion of bound water across the subdomains (e.g., between the slab and mantle wedge) and sublayers (e.g., between basaltic and gabbroic oceanic crusts).

Regarding the liquid-state flow of free water, all the boundaries were set to open with a background volume fraction of free water (10^−4^ or 0.01%), except for the base of the hydrated portion of the subducting slab, defined as no free-water flux boundary (Supplementary Fig. 3c). Except for the serpentinized mantle (> 20% hydration), the isotropic peridotite permeability was used for the subducting slab and mantle wedge. The permeability value (0.01 km) was allocated to the lower portion of the gabbroic continental crust (5 km thick) to account for its low permeability^34^. Initial free water content of 10^−4^ was assigned throughout the model subdomains. No rehydration occurred in the subducting slab; however, dehydration and hydration occurred in the mantle wedge.

The governing equations with the aforementioned rheologies were solved numerically using the commercial finite element package COMSOL Multiphysics® (ver. 5.5). The entire model domain was decomposed into 122,955 unstructured triangular elements, the sizes of which ranged from 0.03125 to 4 km (Supplementary Fig. 3d). For an accurate calculation, the hydrated portions of the subducting slab and base of the mantle wedge mostly consisted of ~ 0.6- and ~ 0.2-km-size elements, respectively. Standard streamline and crosswind diffusions were used for the Stokes and energy equations, and further stabilization of the isotropic diffusion was applied to the bound and free-water transport equations. The fully coupled PARDISO solver was used with the generalized alpha method for time stepping. The model calculation was run for 17 Myr, the period after subduction initiation, by activating the velocity in the subdomain of the subducting slab.

### Supplementary Information

Supplementary Information.

## Data Availability

Data used in this study can be found in the published literatures and cited references. All numerical model results used in this study are available on an open repository, Zenodo: https://doi.org/10.5281/zenodo.7582206.
