# Supplementary material for: Understanding subduction infancy to mature subduction in Southwest Japan via the self-consistent formation of a weak slab interface
Source: Sci Rep. 2023 Dec 5;13:21425. doi: 10.1038/s41598-023-48746-6 (PMC10697977; doi:10.1038/s41598-023-48746-6)
Supplement: Supplementary file 1 — Supplementary Information. [file 41598_2023_48746_MOESM1_ESM.docx]

Supplementary Information

Understanding subduction infancy to mature subduction in Southwest Japan

via the self-consistent formation of a weak slab interface

Changyeol Lee^1^ and YoungHee Kim^2,^*

^1^Department of Earth System Sciences, Yonsei University

50 Yonsei–ro Seodaemun–gu, Seoul, Republic of Korea 03722

^2^School of Earth and Environmental Sciences, Seoul National University

1 Gwanak–ro, Gwanak–gu Seoul, Republic of Korea 08826

^*^Correspondence to: younghkim@snu.ac.kr


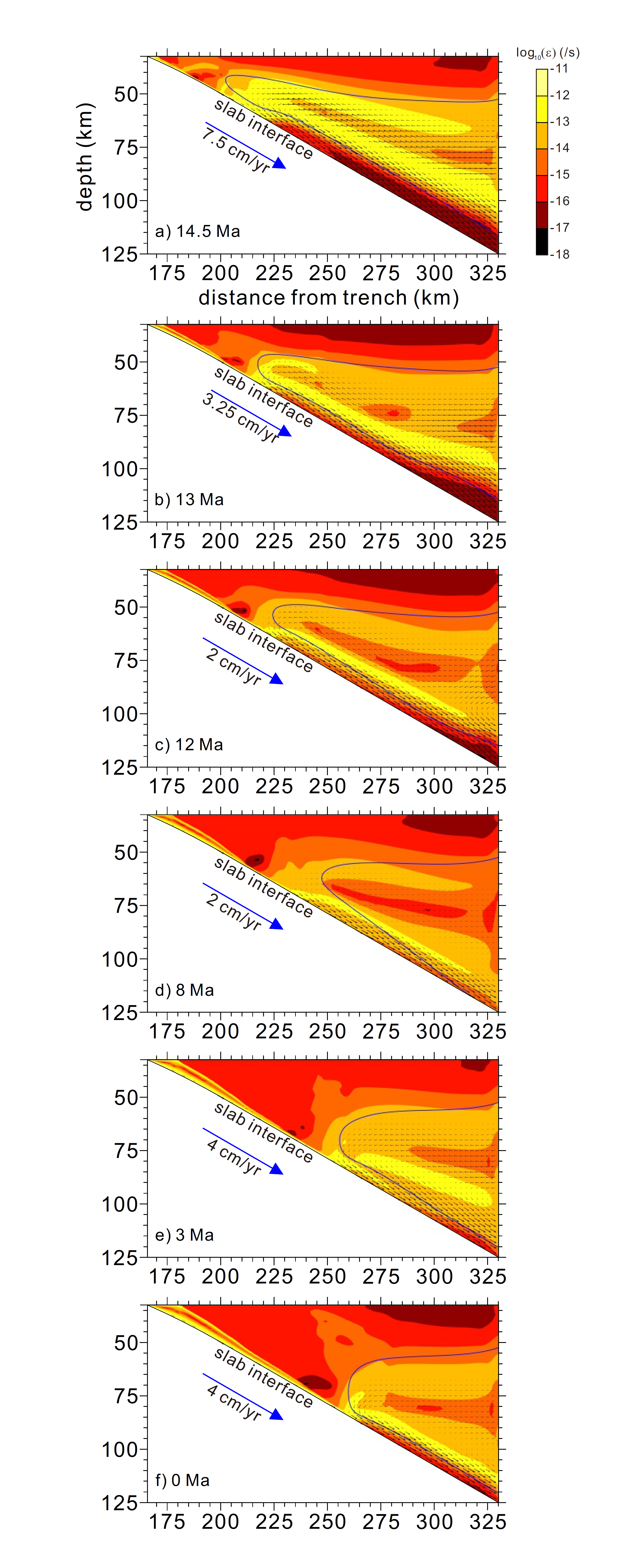


Supplementary Fig. 1. Distribution of strain rate in the mantle wedge obtained from the reference experiment. a–f. Snapshots of the distribution of strain rate shown in Figs. 2–4. The blue contour line in the mantle wedge indicates a temperature of 1000 °C.

**Role of dynamic pressure in the formation of a weak hydrous layer**

We here evaluated the effect of dynamic pressure of the solid flow on the free-water transport by activating the dynamic pressure term in equation 10 shown in **Methods**. To minimize the influence of pressure singularity at the mantle wedge tip, the dynamic pressure at the tip and its neighboring mantle (up to ~5 km away from the tip) was not considered. Other model parameters were the same as those used in the reference experiment.

The results showed that dynamic pressure created complicated flow of free water along the weak hydrous layer and upper mantle beneath the Moho, as shown in 14.5, 13, and 12 Ma, but the spatiotemporal formation of the cold nose in the forearc mantle and the weak hydrous layer at the slab interface was similar to that observed in the reference experiment (Supplementary Figs. 2a1–a3, b1–b3, and c1–c3). After 12 Ma, the impact of the dynamic pressure on the free-water transport was reduced as the subduction rate decreased (2 cm/yr); both the free-water transport and the spatiotemporal formation of the cold nose in the forearc mantle and weak hydrous layer were very similar to those observed in the reference experiment (Supplementary Figs. 2a4, b4, and c4). As the subduction rate increased again, the impact of the dynamic pressure on the free-water transport became intensified at the base of the mantle wedge where the weak hydrous layer broke down as well as along the upper mantle beneath the Moho (Supplementary Figs. 2a5–a6, b5–b6, and c5–c6). In detail, the expelled free water generated by the breakdown of the weak hydrous layer was deflected toward the forearc, forming a narrow channel above the weak hydrous layer, then ascended almost vertically through the mantle wedge, and was slightly deflected toward the forearc beneath the Moho. At 0 Ma, the amount of the free water escaped across the Moho is ~18.4 ton/yr per 1 m along the trench, only ~8.9 % larger than that of the reference experiment. Regardless of the observed complicated fluid flow, our results demonstrated that the impact of dynamic pressure was minimal. Furthermore, the model shows features that were consistent with those observed in the reference experiment, such as (1) spatiotemporal evolution of the cold nose and the weak hydrous layer at the slab interface and (2) present-day free-water transport along the layer as well as the sub-arc slab and flux melting.


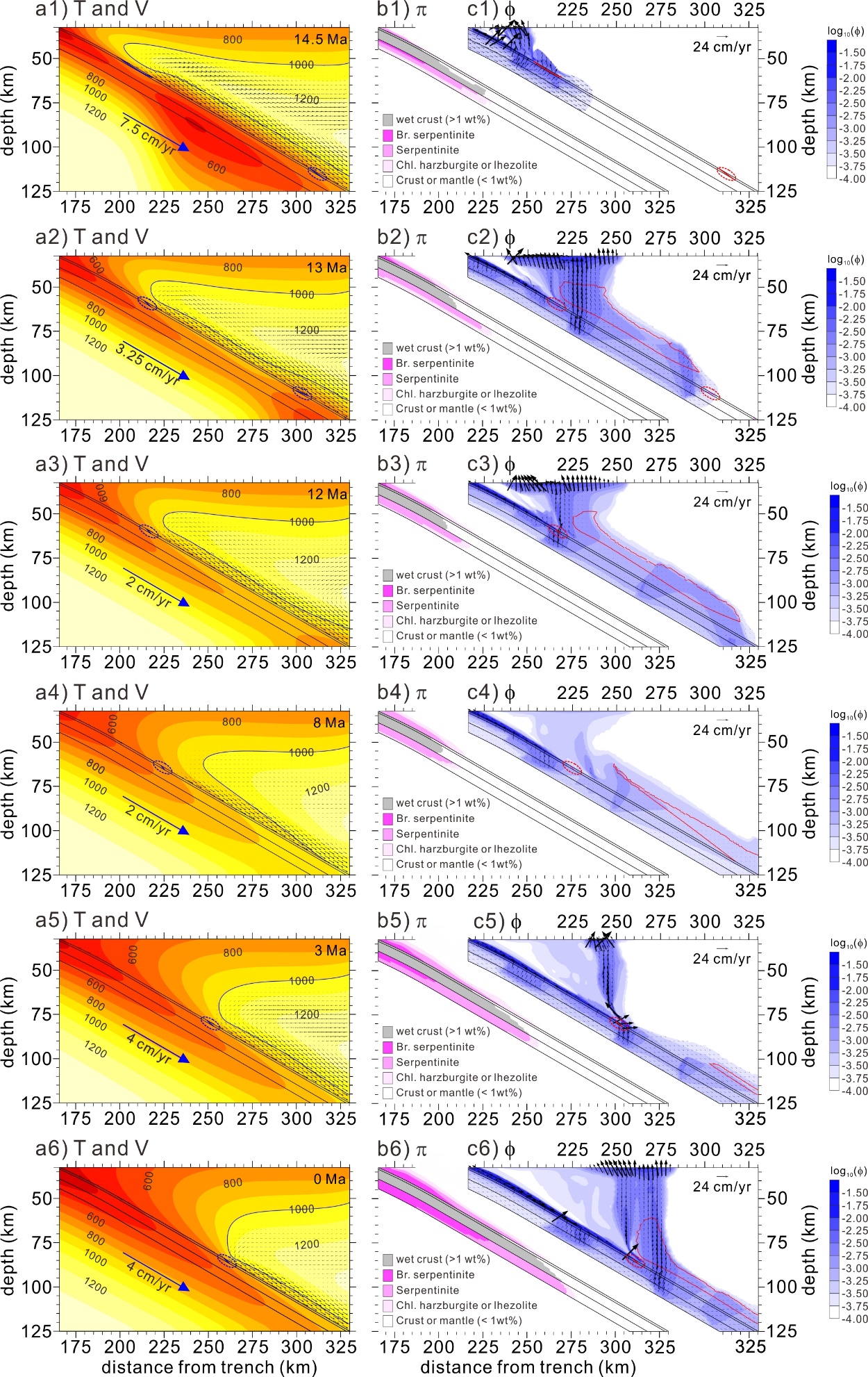


Supplementary Fig. 2. Model results obtained from the reference experiment except for activation of the dynamic pressure term in 14.5, 13, 12, 8, 3, and 0 Ma. a1–a6 Distributions of temperature (T) and velocity (V). Black lines indicate boundaries of the basaltic oceanic crust, the gabbroic oceanic crust, and the hydrated portion of the lithospheric mantle. The magenta lines in the basaltic crust indicate wet basalt solidus fixed at 750 °C. b1–b6 Distributions of major hydrous mineral phases (π) in the lithospheric mantle and “wet” basaltic and gabbroic oceanic crusts (bound water in hydrous minerals > 1 wt%). c1–c6 Distributions of free water (φ) and their velocities ($\boldsymbol{V}_{\boldsymbol{f}}$) in the basaltic and gabbroic crusts and the hydrated portions of the lithospheric mantle and mantle wedge. The red line in the basaltic oceanic crust enclosed by the dashed ellipse indicates wet basalt solidus fixed at 750 °C. The red contour line in the mantle wedge indicates the potential flux melting zone.


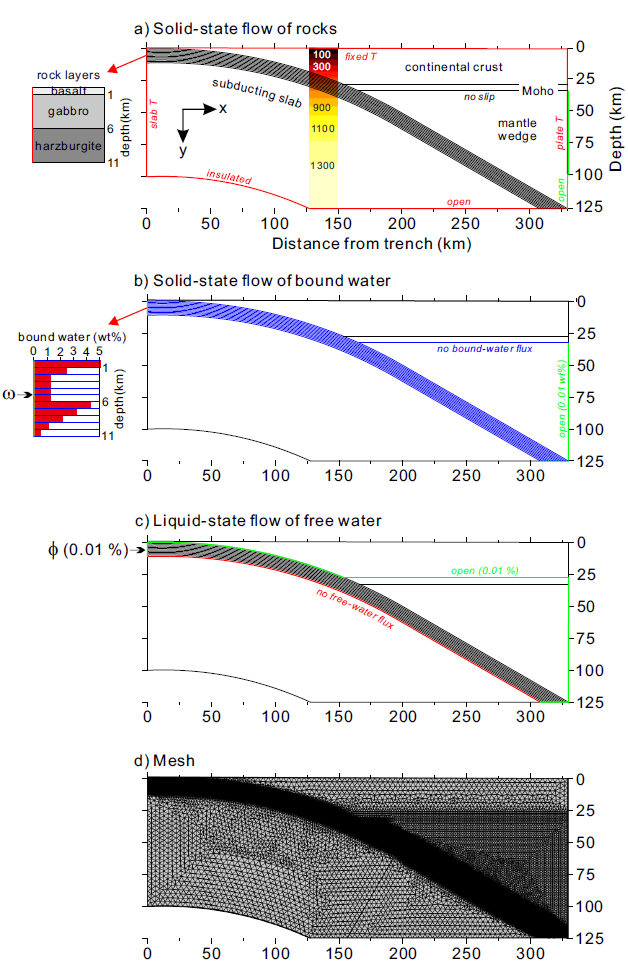


Supplementary Fig. 3. Geometry, boundary conditions, and mesh used in the numerical models. a Boundary conditions for the solid-state flow of rocks with the initial temperature distribution (°C). b Boundary conditions for the solid-state flow of bound water. The depth-dependent distribution of the bound water (ω) is incorporated into the subducting slab through the trench-side vertical boundary. c Boundary conditions for the fluid flow of free water (φ). A 5-km thick low permeability layer of the gabbroic continental crust is prescribed to the lower continental crust with the open boundary at its top. d Mesh consisting of unstructured triangular elements with refinements of the hydrated portion of the subducting slab and base of the mantle wedge.


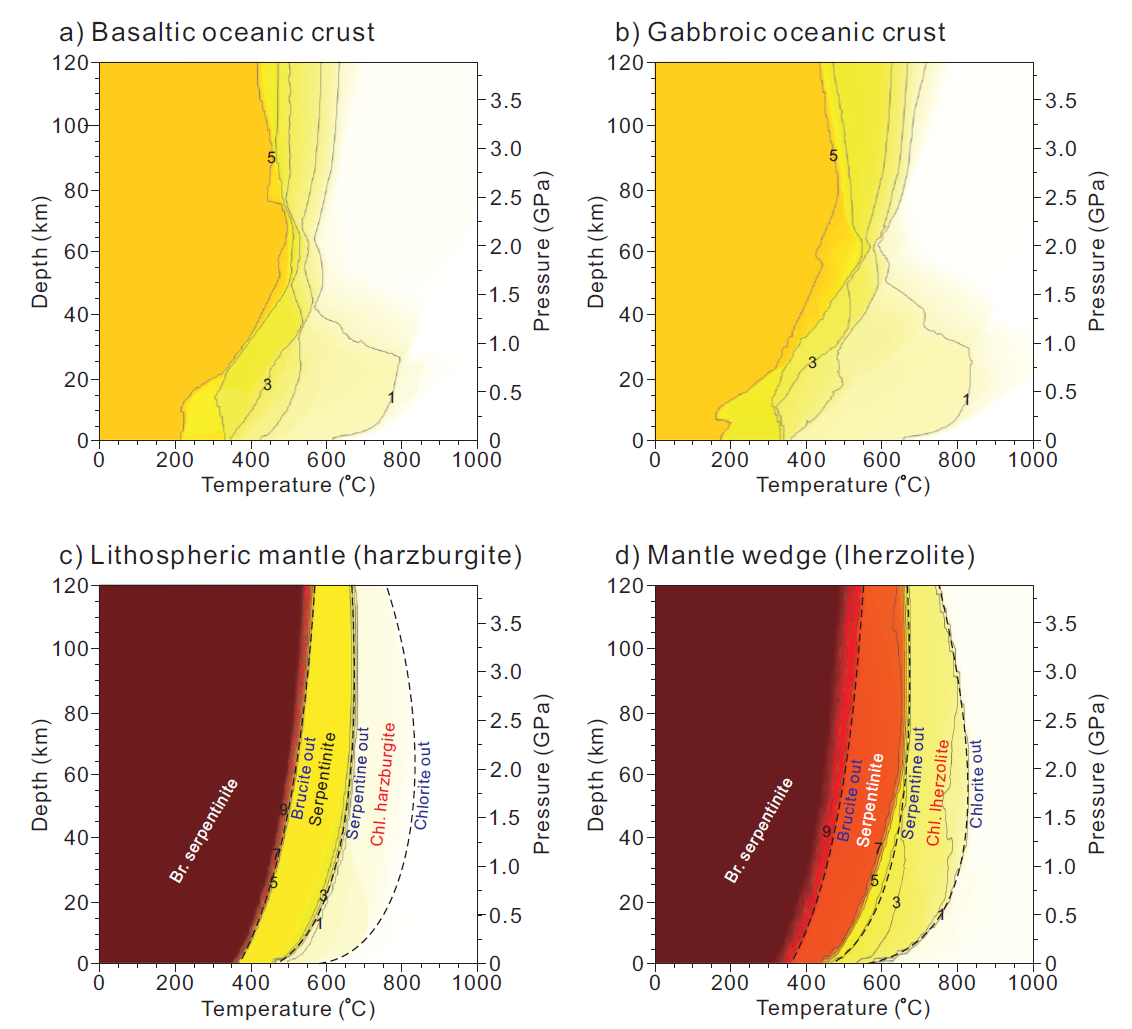
Supplementary Fig. 4. Phase diagrams showing bound-water solubilities for the basaltic oceanic crust, gabbroic oceanic crust, lithospheric mantle, and mantle wedge. a Bound-water solubility of the basaltic oceanic crust of the subducting slab. The numbers indicate water solubilities (wt%). b Bound-water solubility of the gabbroic oceanic crust of the subducting slab. c Bound-water solubility of the lithospheric mantle of the subducting slab represented by residual harzburgite. Br. serpentinite, serpentinite, and chl. harzburgite correspond to brucite-bearing serpentinite, serpentinite, and chlorite-bearing harzburgite, respectively. The black dashed lines represent brucite-, serpentine-, and chlorite-out phase boundaries. d Bound-water solubility of the mantle wedge (lherzolite). All panels were adapted under CC BY-NC 4.0 from Fig. 2^ref.1^.

**Supplementary Table 1. Model parameters**

| **Parameters for the solid rocks** | |  |
| --- | --- | --- |
| Solid (rock) density $\rho_{s}$ (kg m^-3^) | 3300 |  |
| Specific heat at constant pressure *C_p_* (J kg^-1^ K^-1^) | 1000 |  |
| Thermal conductivity *k* (W m^-1^ K^-1^) | 3.0 |  |
| Domain depth *D* (m) | 125×10^3^ |  |
| Thermal diffusivity $\kappa$ (m^2^ s^-1^) | 9.0909×10^-7^ |  |
| Mantle temperature difference $\text{∆T}$ (K) | 1350 |  |
| Net mantle adiabat $\bar{T}$ (K km^-1^) | 0.35×y |  |
| Radiogenic heat production *H* (J kg^-1^ s^-1^) |  |  |
| Oceanic crust (basaltic and gabbroic crusts) | 27×10^-12^ |  |
| Continental crust | 525×10^-12^ |  |
| Mantle | 1.5×10^-12^ |  |
| **Parameters for shear viscosity** | |  |
| Background mass fraction of bound water *ω_r_* (∙) | 10^-4^ |  |
| Shear modulus *μ* (Pa) | 8.0×10^10^ |  |
| Pre-exponent factor *A*   Olivine, diffusion creep $A_{ol,dif}$ (s^-1^)  Olivine, dislocation creep $A_{ol,dis}$ (s^-1^)  Serpentine, dislocation creep $A_{serp,dis}$ (s^-1^) | 8.7×10^15^  3.5×10^22^  4.474×10^-38^ |  |
| Burgers vector *b* (m) | 5×10^-10^ |  |
| Grain size *d* (m) | 2×10^-3^ |  |
| Grain size exponent *m* (∙) Stress exponent *n*  Olivine, dislocation creep $n_{ol}$ (∙)  Serpentine, dislocation creep $n_{serp}$ (∙) | 2.5  3.5  3.8 |  |
| Activation energy *E*  Olivine, diffusion creep $E_{ol,dif}$ (J mol^-1^)  Olivine, dislocation creep $E_{ol,dis}$ (J mol^-1^)  Serpentine, dislocation creep $E_{serp,dis}$ (J mol^-1^)  Activation volume *V*  Olivine, diffusion creep $V_{ol,dif}$ (m^3^ mol^-1^)  Olivine, dislocation creep $V_{ol,dis}$ (m^3^ mol^-1^)  Serpentine, dislocation creep $V_{serp,dis}$ (m^3^ mol^-1^) | 300×10^3^  540×10^3^  8.9×10^3^  6.0×10^-6^  2.0×10^-5^  3.2×10^-6^ |  |
| Reference viscosity $\eta_{0}$ at 125 km depth (Pa·s) | 7.8053×10^19^ |  |
| ^1^Activation function of serpentine viscosity *f(*$\omega$*)* (∙) | 2000 at$\omega$ = 0 500 at $\omega$ = 1 wt% 1 at $\omega$ > 2.153 wt% |  |
| **Parameters for bound and free water** | |  |
| Free-water density $\rho_{f}$ (kg m^-3^) | 1000 |  |
| Free-water viscosity $\eta_{f}$ (Pa s) | 1 |  |
| Artificial diffusivity of free water $\kappa_{\phi}$ (m^2^ s^-1^) | 9.0909×10^-9^ |  |
| Artificial diffusivity of bound water $\kappa_{\omega}$ (m^2^ s^-1^) | 9.0909×10^-9^ |  |
| Gravitational acceleration $g$ (m s^-2^) | 9.81 |  |
| Background volume fraction of free water (porosity) $\phi_{0}$ (∙) | 10^-4^ |  |
| Geometrical factor of grain $C_{d}$ (∙) | 270 |  |
| Dehydration length *l* (km) | 1.25 |  |

Supplementary Table 2. Modeled bulk compositions and Perple_X activity models

| **Modeled bulk compositions** | | | | | | | | | | | | |
| --- | --- | --- | --- | --- | --- | --- | --- | --- | --- | --- | --- | --- |
| **Lithology** | **SiO_2_** | **TiO_2_** | **Al_2_O_3_** | **FeO** | **MnO** | | **MgO** | **CaO** | **Na_2_O** | **K_2_O** | **H_2_O** | **CO_2_** |
| Basalt^2^ | 50.6 | 1.5 | 15.7 | 10.6 |  | 7.6 | | 11.1 | 2.6 | 0.2 | 5.3 | 0.0 |
| Gabbro^2^ | 50.6 | 0.9 | 16.1 | 6.2 |  | 9.2 | | 12.5 | 2.8 | 0.1 | 5.2 | 0.0 |
| Harzburgite^3^ | 43.85 | 0.01 | 0.65 | 7.87 |  | 46.58 | | 0.50 | 0.01 |  | 12.0 | 0.0 |
| DMM^2^ | 44.7 | 0.1 | 4.0 | 8.2 |  | 38.7 | | 3.2 | 0.2 | 0.0 | 12.0 | 0.0 |

| **Perple_X activity models^4^** | | |
| --- | --- | --- |
| **Abbreviation** | **Mineral** | **Solution Model** |
| Anth | Fe-Mg amphibole | ideal |
| Atg | antigorite | ideal |
| B | brucite | ideal |
| Bio(HP) | biotite | Powell and Holland [1999] |
| Carp | carpholite | ideal |
| Chl(HP) | chlorite | Holland et al. [1998] |
| Chum | clinohumite | ideal |
| Cpx(HP) | clinopyroxene | Holland and Powell [1996] |
| Ctd(HP) | chloritoid | White et al. [2000] |
| Feldspar | feldspar | Fuhrman and Lindsley [1988] |
| GlTrTsPg | Na-Ca amphibole | Wei and Powell [2003] and White et al. [2003] |
| Gt(HP) | garnet | Holland and Powell [1998] |
| O(HP) | olivine | Holland and Powell [1998] |
| Omph(HP) | clinopyroxene | Holland and Powell [1998] |
| Opx(HP) | orthopyroxene | Holland and Powell [1998] |
| Pheng(HP) | K-white mica | “Parameters from THERMOCALC” |
| Pl(h) | plagioclase | Newton et al. [1980] |
| San | K-feldspar | Thompson and Hovis [1979] |
| Sp(HP) | spinel | Holland and Powell [1998] |
| St(HP) | staurolite | “Parameters from THERMOCALC” |
| T | talc | ideal |

^2^Hacker (2008)

^3^Ishii et al. (2019) after excluding the Cr_2_O_3_ composition.

^4^Detailed references can be found at https://www.perplex.ethz.ch/PerpleX_solution_model_glossary.html (accessed on March 1, 2022)

**References**

1. Lee, C. & Kim, Y. Role of warm subduction in the seismological properties of the forearc mantle: An example from southwest Japan. *Science Advances* **7**, eabf8934, doi:10.1126/sciadv.abf8934 (2021).
2. Hacker, B. R. H2O subduction beyond arcs. *Geochemistry, Geophysics, Geosystems* **9**, doi:10.1029/2007gc001707 (2008).
3. Ishii, T., Kojitani, H. & Akaogi, M. Phase Relations of Harzburgite and MORB up to the Uppermost Lower Mantle Conditions: Precise Comparison With Pyrolite by Multisample Cell High-Pressure Experiments With Implication to Dynamics of Subducted Slabs. *Journal of Geophysical Research: Solid Earth* **124**, 3491-3507, doi:10.1029/2018JB016749 (2019).
